# The relationship between outflow resistance and trabecular meshwork stiffness in mice

**DOI:** 10.1038/s41598-018-24165-w

**Published:** 2018-04-11

**Authors:** Ke Wang, Guorong Li, A. Thomas Read, Iris Navarro, Ashim K. Mitra, W. Daniel Stamer, Todd Sulchek, C. Ross Ethier

**Affiliations:** 1 Department of Biomedical Engineering, Georgia Institute of Technology/Emory University, Atlanta, Georgia 30332 United States of America; 2 Department of Ophthalmology, Duke University, Durham, North Carolina 27708 United States of America; 3 School of Pharmacy, University of Missouri-Kansas City, Kansas City, Missouri 64110 United States of America; 4 George W. Woodruff School of Mechanical Engineering, Georgia Institute of Technology, Atlanta, Georgia 30332 United States of America

## Abstract

It has been suggested that common mechanisms may underlie the pathogenesis of primary open-angle glaucoma (POAG) and steroid-induced glaucoma (SIG). The biomechanical properties (stiffness) of the trabecular meshwork (TM) have been shown to differ between POAG patients and unaffected individuals. While features such as ocular hypertension and increased outflow resistance in POAG and SIG have been replicated in mouse models, whether changes of TM stiffness contributes to altered IOP homeostasis remains unknown. We found that outer TM was stiffer than the inner TM and, there was a significant positive correlation between outflow resistance and TM stiffness in mice where conditions are well controlled. This suggests that TM stiffness is intimately involved in establishing outflow resistance, motivating further studies to investigate factors underlying TM biomechanical property regulation. Such factors may play a role in the pathophysiology of ocular hypertension. Additionally, this finding may imply that manipulating TM may be a promising approach to restore normal outflow dynamics in glaucoma. Further, novel technologies are being developed to measure ocular tissue stiffness *in situ*. Thus, the changes of TM stiffness might be a surrogate marker to help in diagnosing altered conventional outflow pathway function if those technologies could be adapted to TM.

## Introduction

Previous studies have indicated that trabecular meshwork (TM) stiffness may be related to aqueous humor outflow resistance^1–4^. Specifically, using atomic force microscopy (AFM), TM stiffness was found to be dramatically increased in human glaucomatous eyes compared to that in normal eyes, which was hypothesized to be associated with dysregulation of the extracellular matrix (ECM) observed in glaucoma^1^. Further, in normal and glaucomatous human eyes, we have recently reported an association between outflow facility and TM stiffness as deduced by OCT imaging and numerical modeling^4^. In addition, increased stiffness of TM cells and TM ECM has been reported after exposure to the glucocorticoid dexamethasone (DEX) in cultured human TM cells or in rabbit eyes^5^. Clinically, glucocorticoid exposure can lead to steroid-induced ocular hypertension, which can in turn lead to steroid-induced glaucoma (SIG). SIG has commonalities with primary open-angle glaucoma, and thus an understanding of TM changes in SIG may shed light on TM dysfunction in glaucoma in general.

These previous findings relating increased TM stiffness to glaucoma are important but also subject to certain limitations. For example, post mortem human glaucomatous eyes have typically been treated with anti-glaucoma medications. Thus, it is possible that measured stiffness differences were not directly related to the pathogenesis of glaucoma, but instead were epi-phenomena associated with medications. In other studies involving DEX exposure, TM stiffness and outflow resistance were not both measured, so it is not known if these two factors are associated. It is thus important to repeat TM stiffness measurement in eyes that have not been exposed to glaucoma medications and where the facility can be measured.

TM anatomy and conventional outflow pathway function in mice are generally similar to those in human eyes^6^. For example, outflow facility in mice responds to compounds that similarly affect outflow facility in human eyes^7^. Although mice do not develop POAG as human do, genetically distinct mouse strains have different IOPs, and different conventional outflow facilities^8^. For example, a previous study has shown that there was a correlation between IOP and outflow facility across three mouse strains (CBA/J, C57BL/6J, and BALB/CJ), with 70% of the variation in IOP being attributable to variation in outflow resistance^9^. Thus, using different strains of mice may provide one way to study the relation of TM stiffness to IOP and outflow facility.

DEX-treated animals have been used in several previous studies to examine the effect of glucocorticoids on IOP, outflow facility (C), or TM stiffness. Raghunathan *et al*. reported that topically-administered DEX for 3 weeks resulted in a 3.5-fold increase in TM stiffness in rabbit eyes^5^. However, no statically significant changes in IOP were observed between the DEX-treated eyes and control eyes. Whitlock *et al*. observed a significant IOP increase after systemic DEX treatment using minipump implantation in mice^10^ which can sustain DEX delivery for up to 30 days. Similarly, Overby *et al*.^11^ demonstrated that DEX-induced ocular hypertension (OHT) in mice mimicked hallmarks of human SIG, and that reduced outflow facility was associated with newly formed ECM in the TM (e.g. increased fibrillar material, basement membrane material, etc.). However, systemic delivery of DEX is undesirable due to non-ocular effects, primarily failure of the animals to thrive and gain body mass, and thus alternative DEX delivery methods are preferred. Agrahari *et al*.^12^ have recently developed a method to deliver DEX to human TM cells using DEX-encapsulated pentablock copolymer-based nanoparticles (DEX-PB-NPs). They showed that DEX was released continuously from the PB-NPs over 3 months, with 50% released within the first 6 weeks. This provides the possibility to deliver DEX locally by injecting DEX-PB-NPs into, or adjacent to, mouse eyes, as a mouse model to study the pathophysiology of steroid-induced OHT.

In view of the above, we were motivated to use mice to further investigate the relationship between TM stiffness and outflow function in a controlled fashion. Those two parameters were investigated both in wild-type strains (C57BL/6 J and CBA/J) and after DEX treatment of a single strain (C57BL/6 J), where DEX was delivered into the subconjunctival/periocular space using a custom polymeric nanoparticle carrier, administered once per week for three weeks. If those two parameters are closely related, it would open new paradigms for IOP control and possibly provide new insights into the pathophysiology of POAG and SIG.

## Results

### Effect of genetic background on outflow facility and TM stiffness

**Figure 1:**
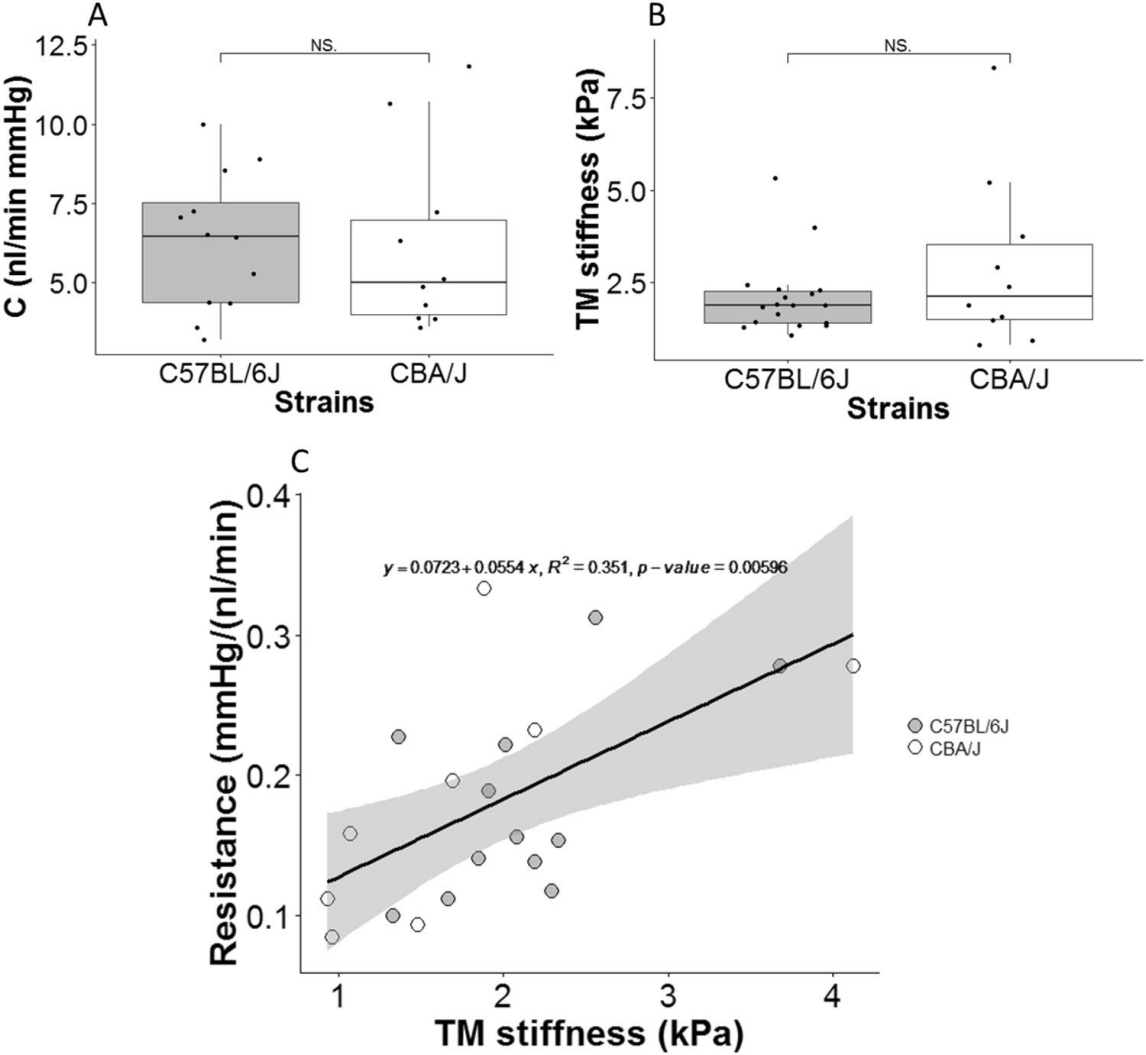
(**A**) Outflow facility (C) and (**B**) TM stiffness for two mouse strains. For each box, the central line represents the median, and the edges of the box are the 25th and 75th percentiles, and whiskers extend to the most extreme data points not considered outliers. Each dot represents the data from one eye per mouse, as described in more detail in the Statistical Analysis section. Due to technical issues, not all measurements in all mice were successful; number of eyes shown for facility plot are n = 12 for C57BL/6 J and n = 10 for CBA/J. For stiffness plot, the respective values are n = 18 and n = 10. NS: Not Significant. (**C**) Cross-plot between outflow resistance (1/C) and TM stiffness, with each data point representing one mouse. The black solid line and equation represent the linear regression of the pooled data. The gray-shaded region shows 95% confidence bounds for the regression. For each mouse, only the data from the OD eye was used, except in cases where the OD eye yielded invalid facility data due to technical issues, in which case data from the OS eye was used. Only mice where both outflow resistance and TM stiffness were measured in the same eyes were included. Number of data points: n = 12 for C57BL/6 J, n = 8 for CBA/J.

We measured outflow facility and stiffness in several pigmented strains of wild-type mice previously reported to have differences in resting IOPs and outflow facility. Stiffness was assayed using an atomic force microscopy-based approach on thawed cyrosections. The outflow facility, a functional measure of the ease of fluid egress from the eye, was not significantly different between C57BL/6J mice and CBA/J mice (mean ± standard deviation: 6.28 ± 2.18 vs. 6.17 ± 2.91 nl/min mmHg; p = 0.692, Fig. 1A). This observation was inconsistent with a previous study^9^ where a statistically significant difference was found. Similarly, average TM stiffness in C57BL/6 J mice was less than in CBA/J mice, but this difference was also not significantly different (mean ± standard deviation: 2.20 ± 1.12 vs. 3.08 ± 3.55 kPa; p = 0.719, Fig. 1B).

Interestingly, we observed a significant correlation between outflow resistance (1/C) and TM stiffness when pooling data from the two strains. We pooled data since there was no significant difference between the two strains in the parameters of the linear regression between resistance and stiffness (p = 0.95 for the slope and p = 0.40 for the intercept, ANCOVA). However, the individual correlations for each strain were not significant (Fig. 1C), which may be explained by the relatively small number of animals in each cohort. Specifically, power analysis suggested that a total of 27 C57BL/6J or 18 CBA/J mice would be needed to reach statistical significance in the relationship between outflow resistance and TM stiffness for each strain individually (α = 0.05, power = 0.9; for C57BL/6J, effect size = 0.43; for CBA/J, effect size = 0.67, GPower software). Overall, this result suggests that there is an inherent relationship between the mechanical properties of TM and outflow resistance in these mice.

### Effects of DEX treatment

**Figure 2:**
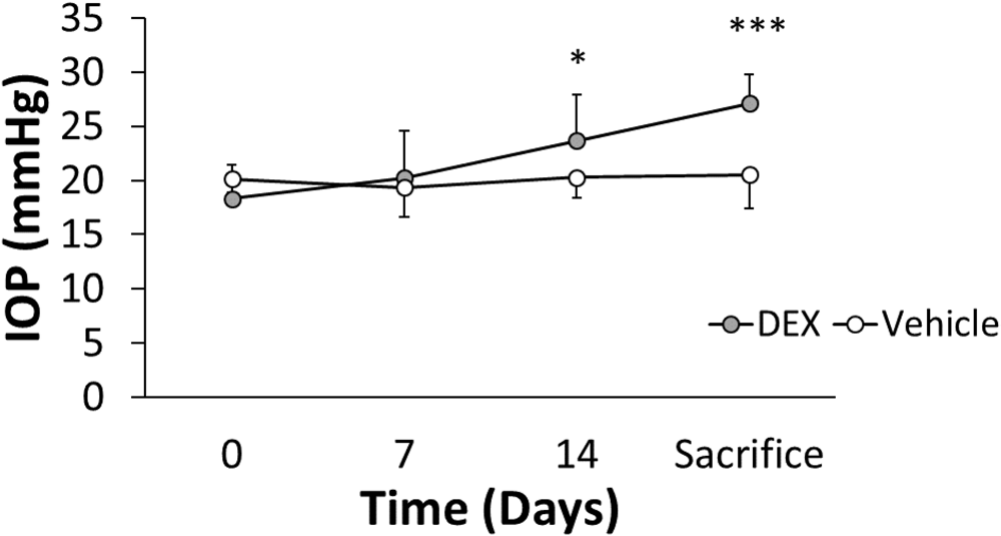
IOP as a function of time for DEX-treated (grey) and vehicle-treated (white) mice averaged over five cohorts. For cohort 1 and 2, DEX or vehicle were injected on day 0 and 14. For cohort 3–5, injections were performed on day 0, 7 and 14. All IOPs were measured immediately before injections. Bars are standard deviation. *p < 0.05, ***p < 0.001. p-values were Benjamini-Hochberg corrected. For each mouse, only the data from the OD eye was used. At day 0, 7 and 14, n = 25 DEX-treated mice and n = 16 vehicle-treated mice. At the day of sacrifice (day 20–40 depending on cohort), n = 24 DEX-treated mice and n = 15 vehicle-treated mice. Two mice were injured during fighting and were euthanized at day 14.

**Figure 3:**
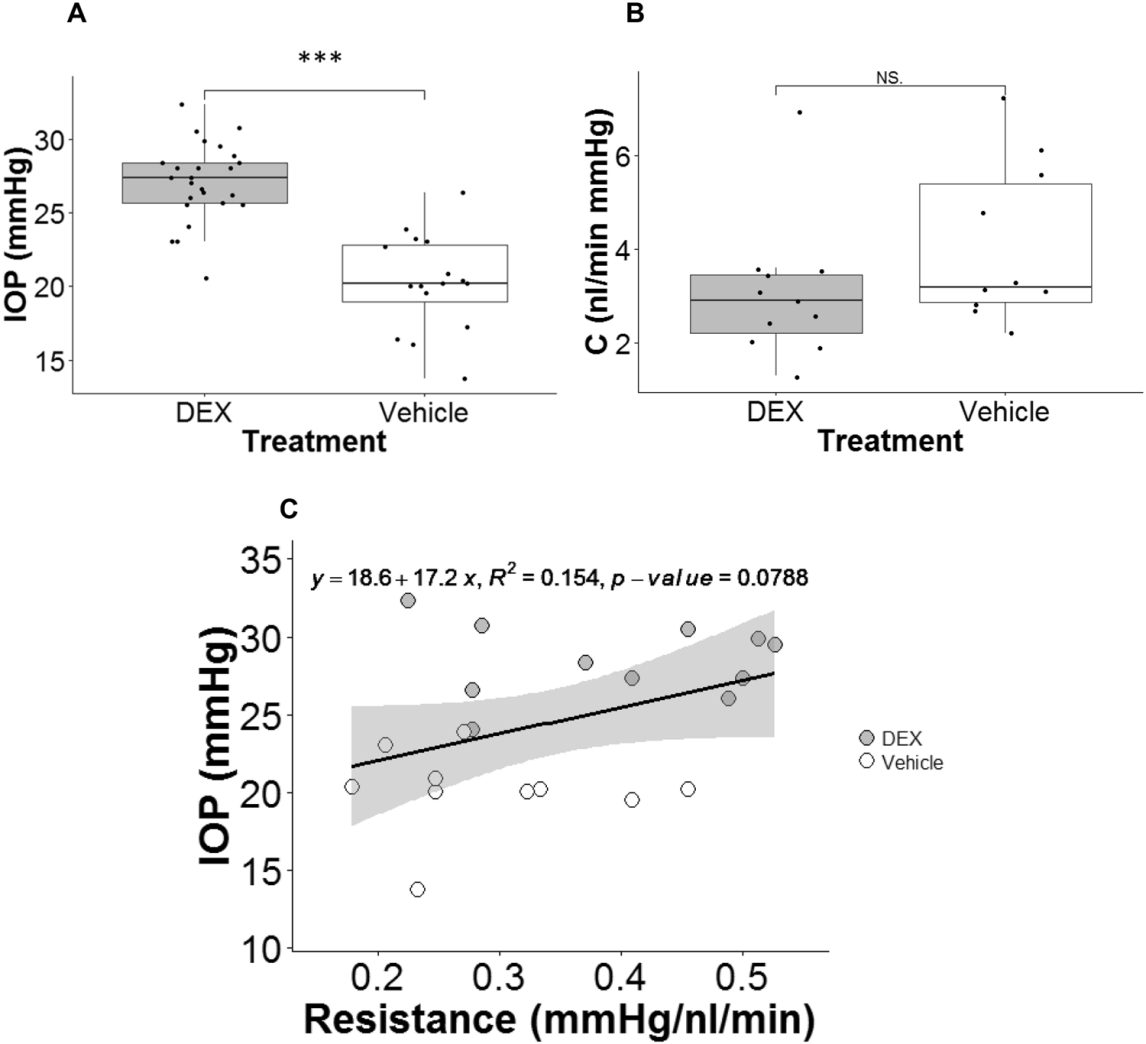
DEX treatment affected IOP and outflow facility. (**A**) Boxplot of IOP for DEX-treated (n = 25) and vehicle-treated mice (n = 16). (**B**) Boxplot of outflow facility (C) for DEX-treated (n = 11) and vehicle-treated mice (n = 10). For each box, the central mark is the median, the edges of the box are the 25th and 75th percentiles, and whiskers extend to the most extreme data points not considered outliers. Each dot represents the data from one eye. (**C**) IOP measured on the day mice were sacrificed plotted as a function of outflow resistance (1/C) for DEX-treated (grey dots, n = 11) and vehicle-treated (white dots, n = 10) mice. The black solid line is the best fit using linear least squares regression. The gray-shaded region shows 95% confidence bounds for the regression. ***p-value < 0.001. NS.: Not Significant. Data in panels B and C are from cohort 3–5.

Local DEX delivery using nanoparticles resulted in significant IOP elevation compared to vehicle-treated animals on day 14 and on the day when mice were sacrificed (Figs 2, 3A). IOP, as measured using rebound tonometry, remained near the baseline level (day 0) in control mice (Fig. 2). On the day mice were sacrificed (typically from day 20–40 depending on cohort), IOP was 27.1 ± 2.7 mmHg (mean ± SD) in DEX-treated mice and 20.5 ± 3.2 mmHg in vehicle-treated mice (p < 0.001, Figs 2, 3A). Further, all DEX-treated mice had increased IOP on the day of sacrifice compared to that on day 0.

The mean facility of DEX mice (strain: C57BL/6 J) was lower than that of control mice (Fig. 3B), but the difference did not reach statistical significance (mean ± standard deviation: DEX: 3.05 ± 1.47 nl/min mmHg, n = 11; Control: 4.09 ± 1.71 nl/min mmHg, n = 10; p = 0.192). The IOP measured before death tended to be negatively correlated with 1/C in the same eye. However, the low R^2^ indicated that only about 15% of the IOP elevation was attributable to the variation in outflow facility, and the relationship was not statistically significant (p = 0.0788; R^2^ = 0.154; Fig. 3C).

**Figure 4:**
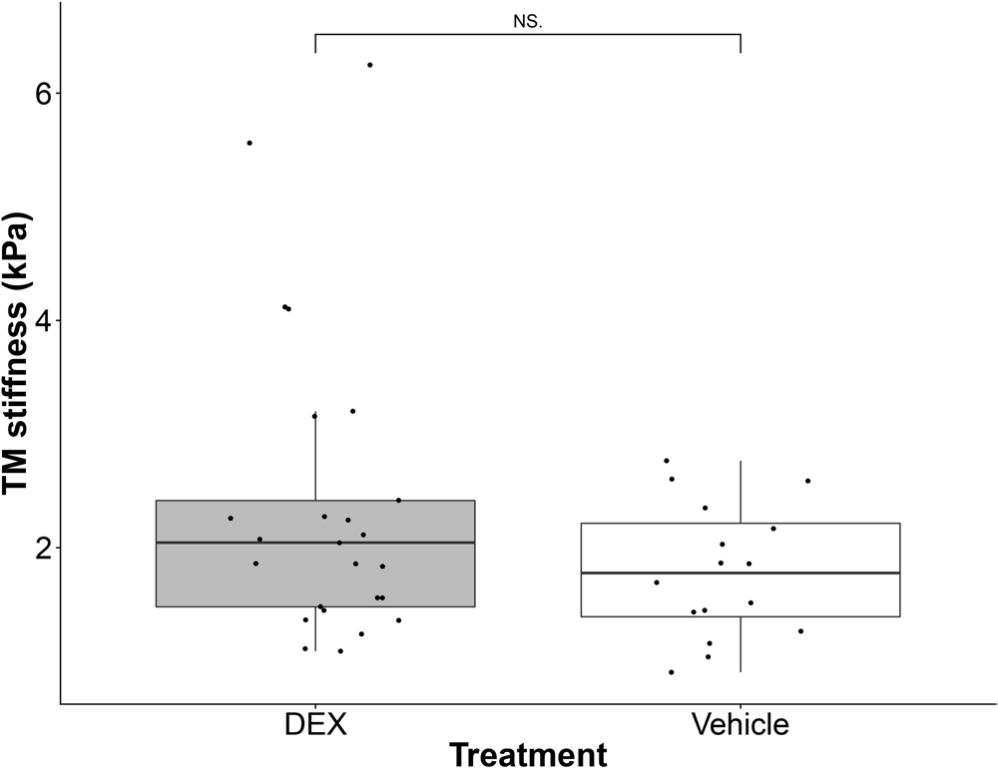
Boxplot of TM stiffness for DEX-treated (n = 25) and vehicle-treated mice (n = 16). For each box, the central mark is the median, the edges of the box are the 25th and 75th percentiles, and whiskers extend to the most extreme data points not considered outliers. Each dot represents the data from one eye. NS: Not Significant.

**Figure 5:**
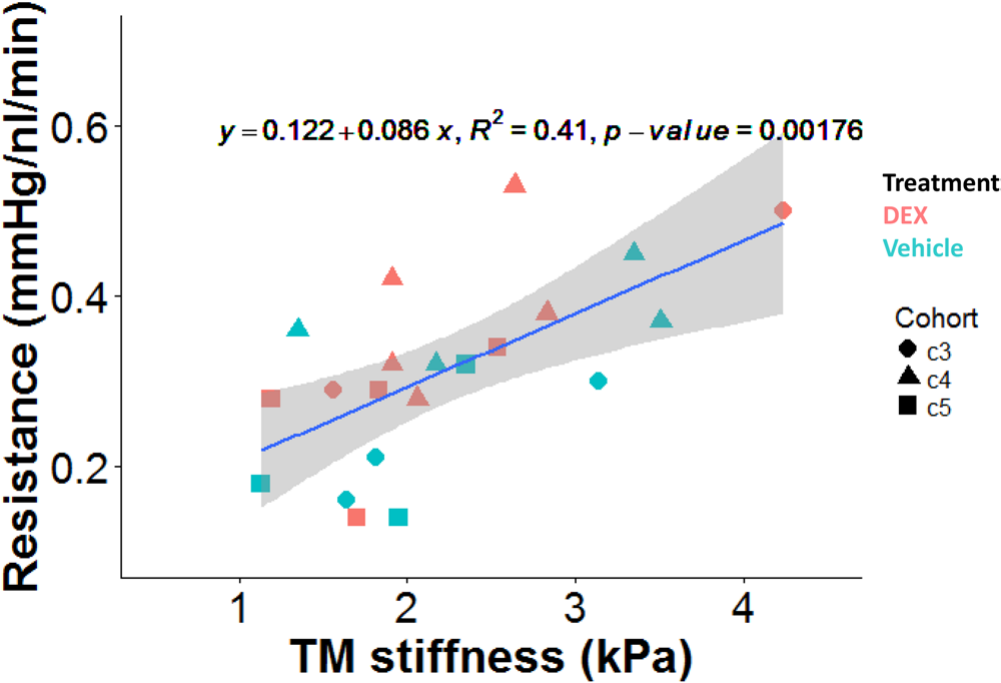
Cross-plot between outflow resistance (1/C) and TM stiffness for DEX-treated (n = 11) and vehicle-treated mice (n = 10). The blue line and equation represent the linear regression of the pooled data. The gray-shaded region shows 95% confidence bounds for the regression. Different shapes represent different cohorts.

**Figure 6:**
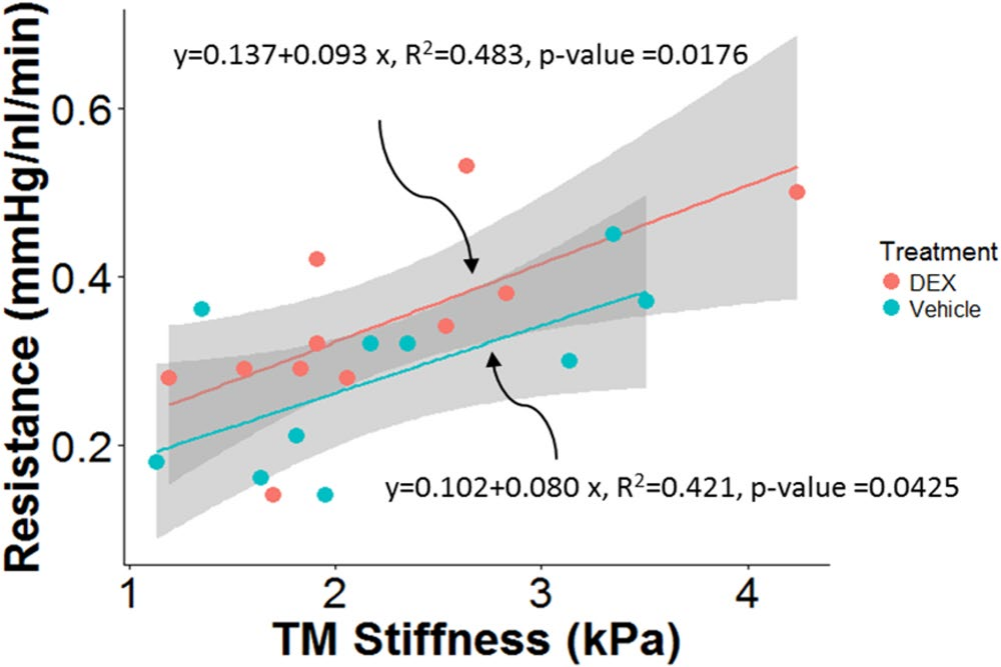
Cross-plot between outflow resistance (1/C) and TM stiffness within each group of mice, with DEX mice shown in red (n = 11) and control mice shown in green (n = 10). The gray-shaded regions show 95% confidence bounds for the regressions.

The TM was stiffer in some of the DEX-treated mice, although not all (Fig. 4). The average TM stiffness in DEX-treated mice was about 20% higher than that in vehicle-treated mice, but this difference did not reach statistical significance (mean ± standard deviation: 2.38 ± 1.31 vs. 1.99 ± 0.91 kPa; p = 0.357). Interestingly, despite the modest differences in facility and TM stiffness (Figs 3B and 4) between the DEX-treated and control mice, there was a positive correlation between resistance and TM stiffness for pooled data of the same mice which was statistically significant (p = 0.002; R^2^ = 0.41; Fig. 5). Mouse cohort, as a covariant, did not have a significant effect on the above correlation (ANCOVA, p = 0.54). Further, the same correlation within each group was also statistically significant (DEX group: R^2^ = 0.483, p = 0.0176; Control group: R^2^ = 0.421, p = 0.0425, Fig. 6). ANCOVA showed that neither the slopes nor the intercepts were significantly different between the two correlations (p for slope = 0.78; p for intercept = 0.09, Fig. 6), justifying performing a single correlation between outflow resistance and TM stiffness using pooled data in Fig. 5.

**Figure 7:**
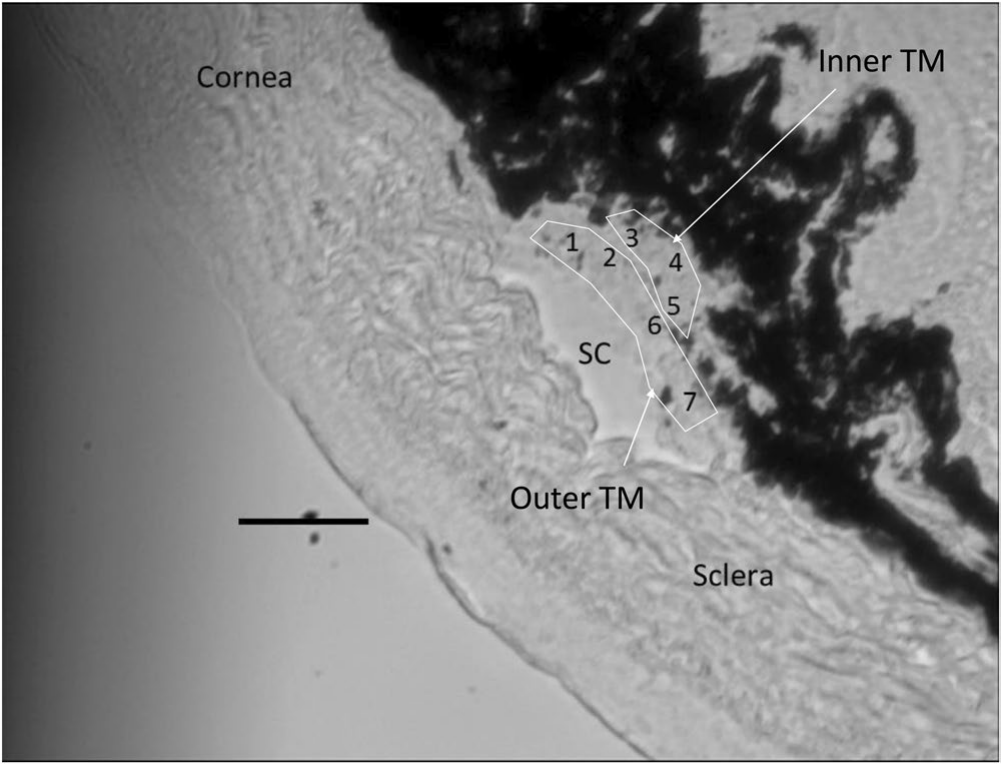
A representative cryosection showing a relatively large TM. Numbers within the TM region indicate individual locations indented by the AFM probe. “Outer” TM and “inner” TM were labeled. Scale bar: 50 µm. Section thickness: 10 µm.

**Figure 8:**
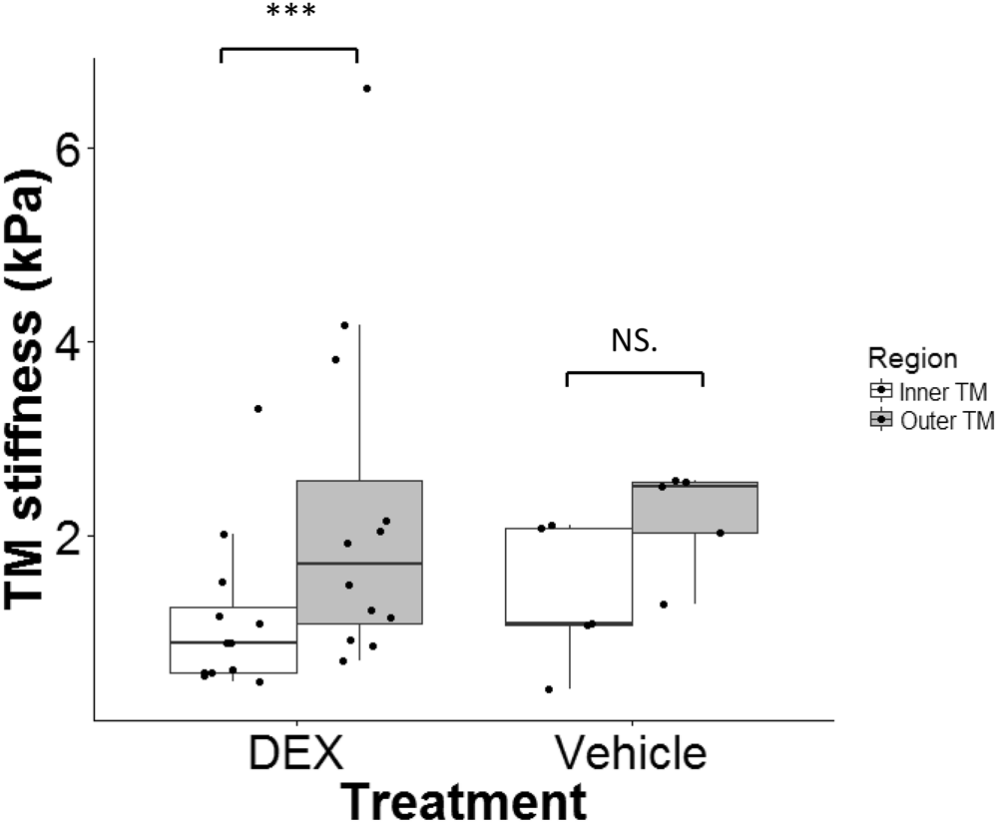
Regional heterogeneity of TM in DEX- (N = 12) or vehicle- (N = 5) treated mice. The outer TM (grey) was stiffer than the inner TM (white) in DEX mice (mean ± SD = 2.26 ± 1.76 kPa for outer TM and 1.14 ± 0.82 kPa for inner TM), but this difference did not reach statistical significance in vehicle mice (2.19 ± 0.55 kPa for outer TM and 1.35 ± 0.73 kPa for inner TM). For each box, the central mark is the median, the edges of the box are the 25th and 75th percentiles, and whiskers extend to the most extreme data points not considered outliers. Each dot represents the data from one animal. ***p < 0.001. NS.: Not Significant.

Within some of the cryosections displaying relatively large TM regions, it was possible to make reliable measurements closer to the SC (“Outer” TM, likely involving the JCT and part of the corneoscleral TM) and further from SC (“Inner” TM, likely including uveal meshwork and part of the corneoscleral TM, Fig. 7). It was consistently observed that the measurements in the outer TM region were stiffer than those in the inner region in both DEX and vehicle-treated mice (Fig. 8), with the difference reaching statistical significance in DEX mice (P < 0.001; paired Wilcoxon test of average inner and outer TM stiffnesses for each mouse; n = 12 mice). This emphasizes the heterogeneity of the TM. A previous study which measured the stiffness of the JCT side of the meshwork demonstrated that low flow (LF) regions of the TM were more rigid than high flow (HF) regions in both normal and glaucomatous TMs^13^. Together, these may suggest that any stiffness differences in HF vs. LF regions of TM may occur closer to the inner wall of SC.

## Discussion

The major finding of this work is that there is a significant correlation between aqueous humor outflow resistance and TM stiffness across two wild-type strains of mice and in DEX-treated mice. This strongly suggests an intrinsic link between the mechanical properties of the TM and the generation of resistance to aqueous humor outflow. These findings are consistent with evidence that Rho-associated protein kinase (ROCK) inhibitors, known to reduce TM actomyosin contractile tone, decrease outflow resistance^14,15^. Interestingly, we have also observed a statistically significant inverse correlation between outflow facility and TM stiffness in a previous study using human tissue from both normal and glaucomatous eyes^4^, consistent with the above results. Taken together, these data suggest commonalities between humans and mice in the TM stiffness-outflow resistance relationship.

The mechanism(s) behind natural variations in TM stiffness, and in the trend towards higher TM stiffness in DEX-treated eyes, remain unclear. It is important to note that we interrogated the stiffness of histologic 10-micron sections, so that most cells within the section had likely been cut during section preparation. It is difficult to unambiguously assign measured stiffness values to a specific TM component in our experiments, but in view of inevitable cell damage sustained during the sample preparation technique, it seems probable that our measurements primarily reflected ECM stiffness. This supposition is consistent with the observations of Overby *et al*., who found that systemic treatment with DEX led to increased ECM in the JCT, including fibrillar material and basement membrane material under the inner wall of Schlemm’s canal^11^. Of course, *in situ*, overall TM stiffness depends on TM cells, the ECM and the interaction between the two^16–21^. It is known that there is a constant mechanobiologic interplay between cells and ECM in soft tissues, so that, for example, TM cell tone can induce ECM reorganization/deposition which may in turn affect TM stiffness^18,22,23^. Thus, the variations in TM stiffness that we observe could have arisen due to cellular-level variations that led to matrix alteration, due to primary variations in ECM composition or density, or both. Better understanding these mechanistic details will be important for developing novel therapies that act on TM stiffness to modulate IOP.

An unexpected observation in our study was that outflow resistance was not significantly related to IOP in the DEX treatment study. There are several possible explanations for this observation, besides the relatively small sample size. Consider Goldmann’s equation:

1 $$Q=C(IOP-EVP)+{Q}_{u}$$

where *Q* is total outflow rate, *Q*_*u*_ is the unconventional outflow rate, EVP is episcleral venous pressure and *C* is the conventional outflow facility. The linear relationship between IOP and outflow resistance (1/C) only holds when three other factors (aqueous humor production rate, episcleral venous pressure and pressure-independent flow) are constant. However, all three factors can differ from animal to animal. Thus, the mathematical relationship between outflow resistance and IOP can vary from mouse to mouse. This may suggest that the TM adapts to “external” factors such as EVP and inflow rate to try to reach a target IOP level.

We also found that, in the genetic background study, neither facility nor TM stiffness showed significant differences between C57BL/6 J and CBA/J mice. This is inconsistent with the results in a previous study, which detected a significant difference in outflow facility between the same two strains^9^. Despite the same trend of facility difference, the actual values from their study were about twofold higher than ours, probably due to different ocular perfusion methodologies and fitting of the flow-pressure curve which have evolved considerably over the past two years^24^.

There are several limitations in this study. First, the DEX concentration was not measured in the DEX-treated eyes in the present study. It is possible that the amount of DEX delivered into the outflow pathway was different from mouse to mouse, which could contribute to variation in the TM stiffness, outflow resistance and IOP between DEX-treated mice. Second, no direct histologic evidence was provided to account for the variation in TM stiffness we observed. Further experiments are needed to address this issue. Third, the effects of the freeze-thaw procedure, inherent in making measurements on thawed cryosections, was not evaluated for its effects on TM mechanical properties. However, no significant ice crystals were observed on the cryosections and previous studies have concluded that the mechanical properties of cryopreserved human arteries and sclera were similar to that obtained in fresh samples^25,26^. Therefore, we expect the effects of freezing on TM mechanical behavior to be small. Nevertheless, it has been reported that freezing-thawing can cause a significant loss of GAG content in articular cartilage, and that this loss was reduced when cryoprotectant was used^27–29^. GAGs are present in the TM and changes in GAG composition have been observed in the TM of glaucomatous eyes^30–32^. Thus, it would be interesting to see whether TM GAG content changes after freezing-thawing, and how this affects TM stiffness, although this would be technically challenging in mouse eyes. Fourth, we did not label the eyes to identify high flow (HF) or low flow (LF) regions; however, we have previously shown that in human eyes, TM stiffnesses were not significantly different between HF and LF wedges^4^. It is interesting to compare this with the findings of Raghunathan *et al*.^33^, who observed that the TM was stiffer in lasered regions vs. non-lasered regions. Although they did not provide direct evidence of high or low flow in these regions, it is reasonable to assume that the lasered region had low flow vs. the non-lasered regions. Their findings thus appear to be opposite to ours; this could be due to the fact that we measured the uveal side of the meshwork in our previous study in human eyes, while Raghunathan *et al*. measured the JCT side, or perhaps the assumption of lasered regions being low-flow is too simplistic. In future studies, it would be worthwhile to identify segmental flow regions in TM by perfusing fluorescent tracers, although the effect of the tracers themselves on TM stiffness would then also need to be investigated.

In summary, this study investigated the role of mechanical properties of TM in outflow function in mouse eyes, showing that outflow resistance is positively and significantly associated with TM stiffness, both in wild-type mice and DEX-treated mice. These data demonstrate that mechanical properties of TM are closely involved in the function of the outflow pathway across a range of conditions. This finding has interesting implications. Importantly, manipulating TM stiffness via mechanisms beyond ROCK inhibition may be a fruitful approach to restore normal outflow dynamics in glaucoma. Further, TM stiffness might be a surrogate marker for conventional outflow pathway function. Novel technologies are being developed to measure ocular tissue stiffness *in situ*^34–37^, and could thus hold promise for future diagnostic benefit in glaucoma if they could be adapted to the TM.

## Methods

### Animals and Overview of Experimental Design

All procedures were approved by the Institutional Animal Care and Use Committee at the Georgia Institute of Technology, and all experiments on living mice were conducted in compliance with the ARVO Statement for the Use of Animals in Ophthalmic and Vision Research. All mice were purchased from the Jackson Laboratory.

We first studied inherent differences in TM stiffness, motivated by the observations that different strains of mice have different resting IOPs and aqueous outflow facilities^8,9^. We chose strains that had previously been characterized and which covered a range of IOPs and facilities, namely CBA/J, C57BL/6 J and BALB/CJ strains. Unfortunately, it proved extremely difficult to measure TM stiffness in BALB/CJ mice, since our technique required the presence of pigmented tissues to identify the location of the TM during stiffness measurements (see below). Thus, a total of 10 CBA/J and 18 C57BL/6 J wild-type mice were used in this study (Age: 10–24 weeks old on the day of sacrifice). For each eye, outflow facility was measured *ex vivo* using the iPerfusion system^24^, after which TM stiffness was measured using our previously developed cryosection-based AFM technique^38^. Due to technical issues, not every measurement was successful, and so the number of reported measurements may be less than the total number of mice. For example, not all measurements of C were technically successful; therefore, the set of mice for which we have data on C is a subset of those for which we have data for E.

To study changes in TM stiffness induced by DEX treatment, 41 C57BL/6 J mice (separated into five cohorts; 25 DEX-treated and 16 vehicle-treated mice; Table 1; Age: 13–19 weeks old on the day of sacrifice, one mouse was 39 weeks old but showed no obvious differences to other mice in the cohort) were used. IOP was recorded before and during DEX treatment. Both outflow facility and TM stiffness were measured in post mortem eyes as described above. All the DEX/vehicle treatments, and IOP and facility measurements for the DEX treatment study were done in the Stamer lab.

**Table 1:** Number of mice, number of valid measurements (i.e. IOP, facility, and TM stiffness) and treated eyes in each cohort for DEX study. OD: right eye; OU: both eyes. IOP: intraocular pressure; C: facility; E: TM stiffness.

Cohort	DEX treatment				Vehicle treatment				Treated eye (s)
	Number of mice	IOP	C	E	Number of mice	IOP	C	E	
1	6	6	0	6	3	3	0	3	OD
2	4	4	0	4	0	0	0	0	OD
3	6	6	2	6	5	5	3	5	OU
4	5	5	5	5	4	4	4	4	OU
5	4	4	4	4	4	4	3	4	OU
Total	25	25	11	25	16	16	10	16	

### DEX Treatment

All PB-NPs were provided by the lab of one co-author (A.K.M.) Information regarding the synthesis of copolymers, formulation of DEX-encapsulated PB nanoparticles and *in vitro* drug release profile have been described in detail in a recent publication^12^. Further, the safety of both PB-NPs and DEX-PB-NPs were evaluated in *in vitro* cell culture system. Briefly, DEX-PB-NPs were formulated by an oil-in-water single emulsion solvent evaporation method as described previously^12^ and stored at -20 °C until further use. The particle size was in the range of 109 ± 3.77 nm. Control PB-NPs (Con-PB-NPs) were prepared with the same approach except that no DEX was added. Nanoparticles were loaded such that 1 mg of DEX-PB-NPs contained 23 µg of DEX, while 1 mg of Con-PB-NPs contained no DEX.

On a typical injection day, DEX-PB-NPs or Con-PB-NPs were dissolved in phosphate buffered saline (PBS) to obtain a DEX-PB-NPs solution or Con-PB-NPs solution at a concentration of 1 mg/20 µl (1 mg DEX-PB-NPs or 1 mg Con-PB-NPs in 20 µl PBS). For each injection, 20 µl of DEX-PB-NPs solution or Con-PB-NPs solution was injected into the superior or inferior subconjunctival/periocular space using 30-gauge needles in DEX and control mice, respectively. Typically, three injections at days 0, 7 and 14 were administered. Importantly, in the first two cohorts of mice, DEX or vehicle was delivered into only the right eyes on days 0 and 14, while the left eyes served as a control. However, we observed a bilateral IOP increase in these animals. Thus, for cohorts 3–5, injections were performed bilaterally, with a subset of mice acting as controls (bilateral Con-PB-NPs injection) and the remaining mice acting being experimental animals (bilateral DEX-PB-NPs injection). Details of mouse numbers in each cohort and the injected eyes are listed in Table 1. A previous *in vitro* study showed that DEX release from the particles typically lasted for 3 months, with about 20% of DEX released in the first 2 days^12^.

### IOP Measurements

IOPs were recorded non-invasively using a commercially available tonometer (TonoLab, TV02, Icare, Vantaa, Finland) in both eyes. Briefly, mice were anesthetized with ketamine (60 mg/kg) and xylazine (6 mg/kg). IOP was immediately measured just as the mice stopped moving (light sleep). Each IOP recorded was the average of six measurements from the same eye. IOPs were obtained right before each injection and on the day when mice were euthanized. All IOP measurements were performed between 10 am to 1 pm.

**Figure 9:**
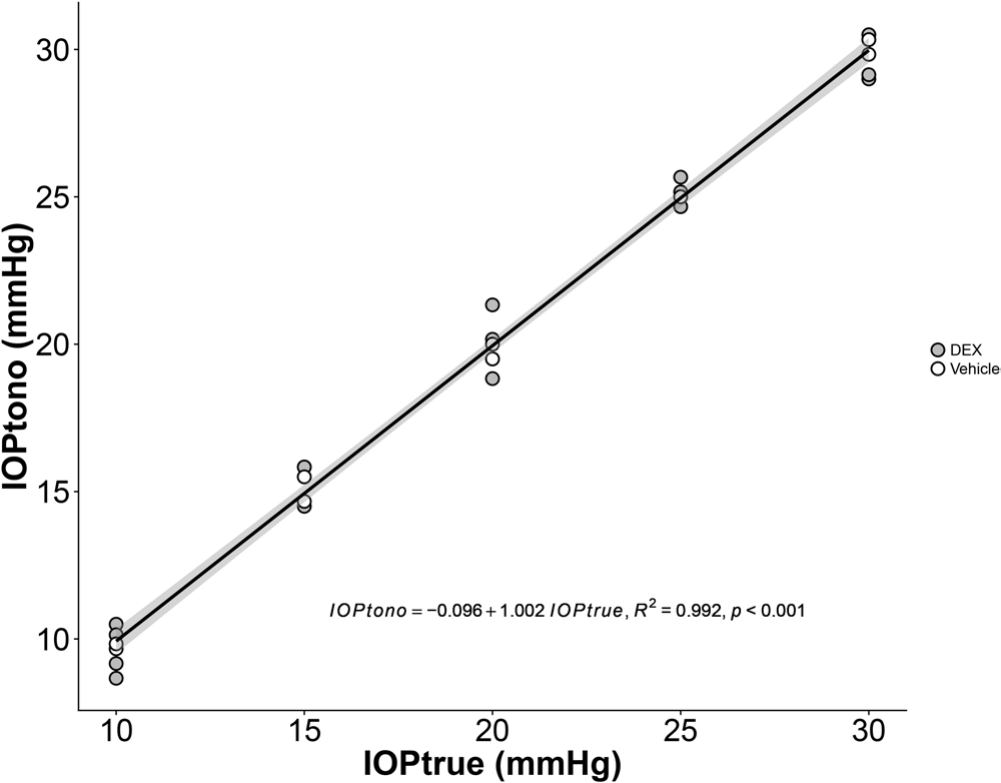
Correlation between IOP measured by tonometer (IOPtono) and set by a reservoir (IOPtrue) in mouse eyes. Each data point refers to a single eye. Grey is for DEX-treated eyes and white is for vehicle-treated eyes. The line is the best fit using linear least squares regression. The gray-shaded region shows 95% confidence bounds to the regression.

We were concerned that the DEX treatment could possibly have affected corneal stiffness, since a previous study showed that DEX treatment softened the cornea in a patient^39^, leading to an artifactual “change” in measured IOP. Thus, the tonometer was calibrated in six eyes of three mice (one control mouse and two DEX-treated mice). Calibration was performed on live, anesthetized animals secured on a platform. The anterior chamber was cannulated with a glass needle (opening ~70 × 80 µm) filled with filtered D-glucose in phosphate-buffered saline (DBG, 5.5 mM). The needle was connected to a pressure transducer (px142–001d5v, Omega Engineering, Stamford, CT) whose output was acquired by a PowerLab system (ML870/P PowerLab 8/30, ADInstruments, Colorado Springs, CO), and then to an adjustable-height reservoir containing filtered ddH2O via 2 stopcocks. IOP was set to either 10, 15, 20, 25, or 30 mmHg by adjusting the reservoir height, and confirmed by the PowerLab system. Before the micropipette was inserted into the anterior chamber, the pressure readings were zeroed to the tear film by placing the needle tip at the same height as it was inside the eye. Tonometer measurements were performed under a microscope to ensure that probe rebounded against the central cornea perpendicularly. Five readings from the tonometer were recorded for each pressure level. For each eye, a linear correlation (equation (2)) between the tonometer-measured pressure (IOPtono) and the true IOP (IOPtrue) was determined from:

2 $$IOPtono=a\cdot IOPtrue+b$$

with slope a, intercept b and R^2^ given in Table 2. Since there was no significant difference in the parameters of the linear regression between treatments (p = 0.70, ANCOVA), all data were pooled together to yield a single regression (Fig. 9).

**Table 2:** Results of the tonometer calibration. R: Pearson Correlation Coefficient.

Groups	Mouse	Eye	a	b (mmHg)	R^2^
Vehicle	1	OD	0.9967	−0.0333	0.9976
		OS	1.0267	−0.5667	0.9997
DEX	2	OD	1.0433	−0.4000	0.9921
		OS	0.9233	1.2333	0.9951
	3	OD	1.0224	1.1524	0.9980
		OS	1.001	0.3429	0.9985

We conclude that DEX treatment did not affect the accuracy of the IOP measurements using the TonoLab tonometer. All IOP values reported in this manuscript were IOPtrue, i.e. IOPs corrected by a single calibration curve as follows (equation (3); note that IOPs are expressed in mmHg in this equation):

3 $$IOPtrue=\frac{IOPtono+0.096}{1.002}$$

### Outflow Facility Measurements

Mice were sacrificed using CO_2_ or isoflurane. Eyes were enucleated using forceps within 5 minutes of death and stored in Dulbecco’s phosphate buffered saline (DPBS, Mediatech Inc, Manassas, VA) at room temperature until use (<10 minutes). Eye perfusion was performed using the previously described iPerfusion system^24^. Briefly, eyes were cannulated with a glass micropipette (outer diameter 70–75 µm, Clunbury Scientific LLC, Bloomfield Hills, MI) under a stereomicroscope using a micromanipulator. LabVIEW software controlling the hardware automatically varied the pressure by adjusting the height of a reservoir connected to the micropipette. Both IOP and flow were recorded in real time. Eyes were perfused at sequential pressures of 4.5, 6, 7.5, 9, 10.5, 12, 13.5, 15, 16.5 and 18 mmHg. Typically, 10 minutes was required at each pressure step to obtain 6 minutes of stable perfusion flow data. During perfusion, the entire eye was submerged in a PBS bath maintained at a temperature of 35 °C. Eyes were perfused with DPBS plus 5.5 mM glucose.

We calculated the pressure-dependent outflow facility by fitting pressure-flow rate data to the following empirical equation:

4 $$Q(P)=C{(\frac{P}{{P}_{r}})}^{\beta }P$$

where *Q* is flow rate (nl/min) measured by a flow sensor, *C* is outflow facility (nl/min mmHg), *β* is a parameter characterizing the nonlinearity of the pressure-flow relationship, $P$ is IOP and ${P}_{r}$ is a reference pressure, taken as 8 mmHg. Using equation (4), values for $C$ and $\beta$ are obtained as fitting outcomes. The reported outflow resistance is the reciprocal of outflow facility.

### TM Stiffness Measurements

**Figure 10:**
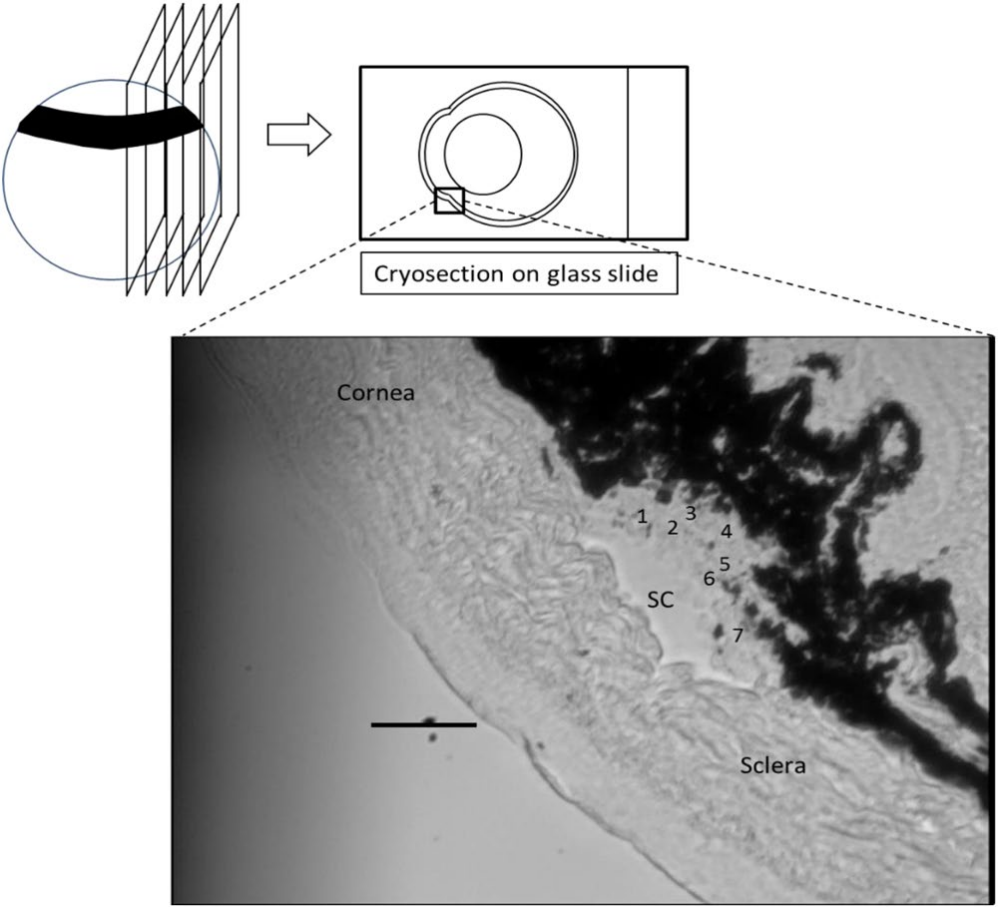
Schematic diagram of the cryosection-based AFM technique. Sagittal cryosections were cut from a frozen eye and mounted to an adhesive glass slide without glue. The bottom figure shows the limbal region of a representative cryosection observed from the AFM bottom camera (SC = Schlemm’s canal). Numbers within the TM region indicate individual locations indented by the AFM probe. Scale bar: 50 µm. Section thickness: 10 µm.

After perfusion, TM stiffnesses were measured using a previously developed AFM technique on cryosections^38^. Briefly, immediately after the glass micropipette was removed from the anterior chamber, a small dab of glue (Superglue, Loctite, Germany) was placed onto the cornea to seal the resulting hole. This ensured that IOP was maintained at a value close to the last perfusion value, so that an open SC lumen was more likely to be found, aiding in TM localization (Fig. 10). Eyes coated with optimal cutting temperature compound (O.C.T.; Tissue-Tek) were then frozen by immersion in 2-methylbutane (Sigma-Aldrich, St. Louis, MI) cooled by liquid nitrogen^40,41^. For each eye, a few 10 µm thick sagittal cryosections from 3 different quadrants were cut on a Microm Cryostar NX70 cryostat (Dreieich, Germany), The eye was mounted and oriented on a cryostat chuck to allow sagittal (anterior to posterior) sections to be cut through the whole globe. Using the cryostat blade, approximately 1.0 mm of tissue was cut away, then 8 to 16 sections were collected. These whole globe sections allowed AFM measurement of two opposing quadrants. For measurement of the third quadrant, the tissue was unmounted, rotated 90° to the plane of section, approximately 1.0 mm of tissue was again cut away, and 8 sections were then harvested. The sections were collected on adhesive slides (Plus gold slide, Electron Microscopy Sciences, Hatfield, PA) and stored for up to 30 min in ice-cold PBS prior to AFM analysis (Fig. 10).

Samples were transferred to an MFD-3D AFM (Asylum Research, Santa Barbara, CA) and kept continuously immersed in PBS during measurements at room temperature. TM stiffnesses were measured following the same protocol we used previously^38^. Specifically, for each cryosection, the TM was first localized as the region between the pigmented ciliary body and the inner wall endothelium of SC (Fig. 10). Multiple locations in the TM region (typically 3–9) were probed by the cantilever and three repeated measurements were conducted at each location. The average from the three measurements was taken as the TM stiffness at that location. TM stiffnesses from all locations within a cryosection were then averaged to obtain the TM stiffness of that cryosection. We typically made AFM measurements on 9 cryosections (collected from 3 different quadrants) from each individual eye, since TM stiffness may be location-dependent. Finally, the mean stiffness of all cryosections was taken as the TM stiffness of the eye. Cantilever probes were modified by attaching a spherical indentor of diameter 10 µm to smooth nanoscale variations in tissue mechanics. For each indentation, the indentation depth was 0.5–1 µm, with a maximum applied force of 7 nN and approach velocity of 8 µm/s. A Hertzian model was used to extract a Young’s modulus stiffness value from the force versus indentation curves. Three force curves were obtained per location, leading on average to 135 in total for each eye (5 locations for each cryosection on average and typically 9 cryosections per eye).

### Statistical Analysis

For each mouse, only the data from the OD eye were used to avoid statistical non-independence effects between the two eyes from one animal^42^. The exception was in animals for which measurements on the right eye yielded invalid facility data due to technical issues, in which case OS was used instead of OD. In cohorts 1 and 2 in the DEX study, only the OD eye was treated; fortunately, all IOP and stiffness measurements were successful in these eyes. Note that facility was not measured in any eyes of any mice from cohorts 1 and 2. For completeness, and for consistency with other studies, we also repeated our analyses by averaging data from both eyes of each mouse, using the same statistical methods. The main conclusions were unchanged when the data were analyzed in this way. We chose to here present data on a one eye per mouse basis, since we were concerned that averaging between eyes of a single mouse could obscure relationships when correlating stiffness and outflow resistance.

The Wilcoxon rank-sum test was used to compare differences in IOP, outflow facility and TM stiffness between groups (C57BL/6 J vs. CBA/J wild-type mice or DEX-treated vs. Vehicle-treated mice). All data was presented as the mean ± standard deviation. To test the correlation between IOP and outflow resistance, and the correlation between outflow resistance and TM stiffness, we used linear regression. Further, ANCOVA was used to investigate whether other factors (e.g. treatment conditions, cohorts) were significant contributors to the changes in outflow resistance (R; version 3.4.1; R Core Team). In those models, the TM stiffness was the independent variable and outflow resistance was the dependent variable. Other factors were treated as covariates. The significance threshold was defined to be 0.05 for all statistical tests.

### Data Availability Statement

The datasets generated during and/or analyzed during the current study are available from the corresponding author on reasonable request.
